# SPR Biosensor Based on Bilayer MoS_2_ for SARS-CoV-2 Sensing

**DOI:** 10.3390/bios15010021

**Published:** 2025-01-04

**Authors:** Talia Tene, Stefano Bellucci, Cristian Vacacela Gomez

**Affiliations:** 1 Department of Chemistry, Universidad Técnica Particular de Loja, Loja 110160, Ecuador; tbtene@utpl.edu.ec; 2 INFN-Laboratori Nazionali di Frascati, Via E. Fermi 54, 00044 Frascati, Italy; bellucci@lnf.infn.it

**Keywords:** surface plasmon resonance, MoS_2_, silicon nitride, SARS-CoV-2, biosensor

## Abstract

The COVID-19 pandemic has highlighted the urgent need for rapid, sensitive, and reliable diagnostic tools for detecting SARS-CoV-2. In this study, we developed and optimized a surface plasmon resonance (SPR) biosensor incorporating advanced materials to enhance its sensitivity and specificity. Key parameters, including the thickness of the silver layer, silicon nitride dielectric layer, molybdenum disulfide (MoS_2_) layers, and ssDNA recognition layer, were systematically optimized to achieve the best balance between sensitivity, resolution, and attenuation. The optimized configuration, consisting of a 45 nm silver layer, a 13 nm silicon nitride layer, 2 MoS_2_ layers, and a 5 nm ssDNA layer, demonstrated superior performance for detecting SARS-CoV-2 in PBS solution. The biosensor exhibited high sensitivity at low viral concentrations, achieving a sensitivity of 375.01°/RIU, a detection accuracy of 0.002, and a quality factor of 38.34 at 1.0 mM SARS-CoV-2 concentration. Performance metrics validated the sensor’s capability for reliable detection, particularly in early-stage diagnostics where timely intervention is critical. Moreover, the biosensor’s linear response to refractive index changes confirmed its potential for quantitative viral concentration analysis. This study underlines the significance of integrating advanced materials, such as MoS_2_ and silicon nitride, to enhance SPR biosensor performance. The findings establish the proposed biosensor as a robust and precise diagnostic tool for SARS-CoV-2 detection, with potential applications in clinical diagnostics and epidemiological monitoring.

## 1. Introduction

The emergence of SARS-CoV-2, the virus responsible for the COVID-19 pandemic, has dramatically affected global public health and economies [1], resulting in widespread disruptions and a substantial loss of life [2]. Early detection and containment efforts have played a critical role in mitigating the impact of the virus. The gold standard for SARS-CoV-2 detection has been reverse transcription polymerase chain reaction (RT-PCR) [3], a molecular diagnostic method that identifies viral RNA with high specificity and sensitivity. However, despite its effectiveness, RT-PCR is limited by long processing times, the need for specialized laboratory infrastructure, and the reliance on trained personnel. These limitations have highlighted the demand for alternative diagnostic tools that are not only rapid and reliable but also accessible for point-of-care use in diverse settings, especially during times of large-scale outbreaks [4].

Biosensors, particularly surface plasmon resonance (SPR) biosensors, have emerged as a promising alternative for viral detection due to their ability to provide real-time label-free analysis of biomolecular interactions [5,6]. SPR biosensors function by measuring changes in the refractive index at a metal–dielectric interface when biomolecules bind to the surface of the sensor [7]. This interaction leads to a detectable shift in the SPR angle, which correlates with the presence and concentration of the target analyte, such as a viral protein or RNA [8]. SPR-based detection systems offer numerous advantages, including rapid response times, high sensitivity, and the potential for reusability, making them suitable for widespread diagnostic applications [9].

Despite these advantages, the sensitivity of SPR biosensors can be further enhanced by incorporating advanced materials that improve surface interactions and signal amplification [10]. Two-dimensional (2D) materials, such as Molybdenum Disulfide (MoS_2_), have shown great potential in this regard. MoS_2_, a transition metal dichalcogenide (TMD) family member, has unique electrical, optical, and mechanical properties that make it particularly well-suited for biosensing applications [11,12]. In its bilayer form, MoS_2_ offers a high surface area, strong light–matter interaction, and excellent biocompatibility, all of which contribute to improved biosensor performance [13]. Specifically, bilayer MoS_2_ can enhance the detection of viral particles by increasing the interaction between the sensor surface and the target biomolecules, such as the spike protein (S protein) or nucleocapsid protein (N protein) of SARS-CoV-2.

Alongside MoS_2_, materials like silicon nitride (Si_3_N_4_) have also been employed in biosensor technologies due to their complementary properties [14]. Silicon nitride is a ceramic material known for its mechanical strength, optical transparency, and biocompatibility, making it an excellent substrate for biosensor platforms [15]. In SPR biosensors, Si_3_N_4_ acts as a dielectric layer that enhances the propagation of surface plasmons, leading to greater sensitivity in detecting small changes in the refractive index. Additionally, the surface of Si_3_N_4_ can be easily functionalized with biomolecules such as antibodies or aptamers, which specifically bind to viral components, thereby improving the selectivity of the biosensor. The combination of bilayer MoS_2_ and Si_3_N_4_ within an SPR biosensor framework offers a novel approach to achieving high sensitivity and specificity in SARS-CoV-2 detection.

Here, we then present an SPR biosensor based on bilayer MoS_2_ for the detection of SARS-CoV-2. By leveraging the high surface area and signal amplification capabilities of bilayer MoS_2_, along with the dielectric properties of Si_3_N_4_, we aim to develop a biosensor that provides high sensitivity and reliable detection of SARS-CoV-2 at different concentrations. The proposed biosensor is designed to detect viral proteins or RNA with minimal sample preparation, making it suitable for rapid point-of-care diagnostics. Furthermore, the integration of these advanced materials into the SPR platform represents a step toward the development of next-generation biosensors capable of addressing current and future viral outbreaks. The results of this study could pave the way for the development of highly sensitive multiplexed detection systems that can be adapted to detect a wide range of pathogens.

## 2. Materials and Methods

### 2.1. Biosensor Configurations

**Figure 1:**
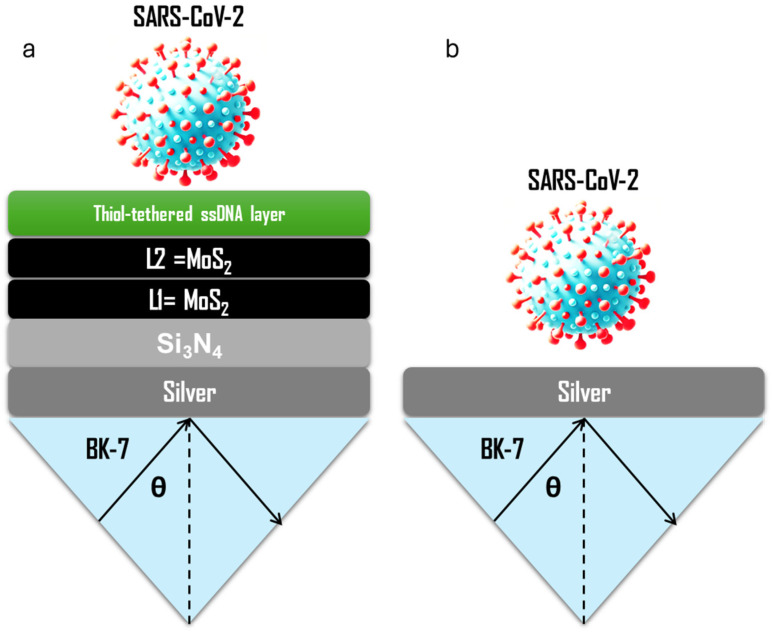
Schematic representation of the proposed SPR Biosensor for detecting the novel coronavirus. (**a**): SPR biosensor based on bilayer MoS_2_; (**b**): Baseline biosensor.

To begin, Figure 1a presents the multilayered biosensor developed in this study, while Figure 1b shows a basic SPR biosensor for comparison. The proposed biosensor integrates a multilayer architecture that incorporates advanced materials such as bilayer MoS_2_ (obtained as the best parameter after optimization) and Si_3_N_4_ to improve sensitivity and specificity for detecting SARS-CoV-2. The uppermost layer in the design consists of a thiol-tethered single-stranded DNA (ssDNA) layer, which is functionalized to selectively bind to SARS-CoV-2 viral RNA [16]. The thiol groups form a stable chemical bond with the sensor surface, ensuring robust attachment of the ssDNA for efficient recognition of viral genetic material [17]. Beneath the functionalized layer, the sensor includes two layers of MoS_2_ (denoted as L1 and L2), which enhance the performance of the biosensor. Additionally, the optical properties of MoS_2_ are expected to contribute to signal amplification, enhancing the ability of the sensor to detect even small changes in the refractive index caused by the binding of viral particles. This is particularly important in applications where low viral loads, such as those present in early-stage infections or asymptomatic individuals, need to be detected with high accuracy.

The Si_3_N_4_ layer serves as a dielectric substrate that further enhances the propagation of surface plasmons generated by the SPR process. The dielectric properties of Si_3_N_4_ ensure that the plasmonic field is well-confined at the sensor surface, improving the sensitivity of the sensor to refractive index changes. Additionally, Si_3_N_4_ provides mechanical stability and optical transparency, making the sensor robust for practical use in clinical or point-of-care settings [18]. At the core of the sensor lies a thin silver layer, which acts as the plasmonic material [19]. When polarized light strikes the silver surface at a specific angle (θ), it excites surface plasmons—coherent oscillations of electrons at the metal–dielectric interface [20]. This plasmonic excitation is highly sensitive to changes in the refractive index near the sensor surface, making silver an ideal material for SPR-based detection. The entire biosensor is built on a BK-7 glass substrate, which ensures structural support and maintains optical compatibility with the SPR detection system.

On the other hand, Figure 1b depicts a simplified SPR biosensor design, which lacks the advanced material enhancements present in the proposed multilayer structure. This design includes only the essential components for SPR detection: a silver layer for plasmon excitation and a BK-7 glass substrate for structural support. The detection mechanism in this configuration is based solely on the interaction of SARS-CoV-2 particles with the silver surface, which induces a shift in the SPR angle. However, the lack of MoS_2_ and Si_3_N_4_ limits the sensitivity and specificity of the sensor.

Table S1 presents the system configurations investigated in the study to determine the most effective configuration for the SPR biosensor used for SARS-CoV-2 detection. The various configurations systematically explore different combinations of materials, including silver (Ag), silicon nitride (Si_3_N_4_, SN), molybdenum disulfide (MoS_2_, MS), and a thiol-tethered single-stranded DNA (ssDNA, T) recognition layer, to evaluate their effects on the performance of the sensor in a phosphate-buffered saline (medium, M) solution.

Table S2 presents the initial material parameters [21,22,23,24,25], including refractive index and thickness, used in the construction of the SPR biosensor incorporating one layer of MoS_2_. With a refractive index of 1.5151, BK-7 glass ensures minimal loss of light as it passes through the prism and reaches the silver layer where surface plasmons are excited. The refractive index of silver is 0.056253 + 4.2760i, where the imaginary part reflects the inherent losses of the material due to absorption. The thickness of the silver layer is 55.0 nm, which is considered ideal for achieving a strong SPR signal while minimizing energy losses. With a refractive index of 2.0394 and a thickness of 5.0 nm, the Si_3_N_4_ layer provides mechanical stability, making the sensor more durable for use in clinical environments.

A single layer of MoS_2_ is used for optimization, with a thickness of 0.65 nm. The refractive index of MoS_2_ is 5.0805 + 1.1723i, reflecting its strong interaction with light and relatively low losses compared to other materials. The refractive index of the ssDNA layer is 1.462 and its thickness of 3.2 nm provides sufficient surface area for the ssDNA molecules to effectively bind to the viral RNA while maintaining a stable and thin profile that minimizes interference with the SPR signal. Finally, phosphate-buffered saline (PBS) solution acts as the surrounding medium in which the biosensor operates, mimicking the physiological conditions necessary for biomolecular interactions. The refractive index of PBS is 1.334.

We further point out that the performance of biosensors in practical applications heavily relies on addressing critical challenges such as selectivity, cross-sensitivity, and false prediction. In our proposed SPR biosensor design, these aspects have been considered and optimized as follows:

- The incorporation of a thiol-tethered ssDNA layer in the sensor design ensures high selectivity for SARS-CoV-2 RNA. The thiol chemistry provides a stable attachment to the underlying surface layer, enhancing durability and reproducibility. Furthermore, the ssDNA sequence is specifically tailored to enable strong and selective hybridization with the target viral RNA, thereby minimizing false interactions and improving the accuracy of detection;
- Cross-sensitivity, which can lead to interference from non-specific biomolecular interactions, is mitigated through two complementary mechanisms. First, the ssDNA layer’s specificity inherently reduces cross-reactivity with non-target molecules. Second, the multilayer structure, which includes Silicon Nitride and MoS_2_, enhances plasmonic confinement. This improved confinement reduces the influence of non-specific interactions on the SPR signal, ensuring high-fidelity detection even in complex biological environments;
- False predictions are minimized by leveraging the high sensitivity (375.01°/RIU) and a quality factor (QF) of 38.34 achieved by our multilayer design. These parameters ensure a robust signal-to-noise ratio, which is critical for distinguishing true target-binding events from noise. Additionally, the functionalized ssDNA layer is engineered to prevent non-specific binding, further reducing the potential for false positives and enhancing the reliability of the biosensor.

These design features collectively ensure that the proposed SPR biosensor achieves a balance of high sensitivity, specificity, and robustness, making it a promising tool for the detection of SARS-CoV-2.

### 2.2. Modeling Approach

We have carried out a numerical analysis to calculate the reflectance curve using TMM and the Fresnel equation; see details about the mathematical model in [26,27]. Then, the total reflection analysis of the N-layer system is obtained as

(1) $$R={\left|\frac{\left(M_{11}+M_{12}q_{N}\right)q_{1}-\left(M_{21}+M_{22}q_{N}\right)}{\left(M_{11}+M_{12}q_{N}\right)q_{1}+\left(M_{21}+M_{22}q_{N}\right)}\right|}^{2}$$

It is noted that for each SPR curve, surface plasmon excitation is identified as a dip in the reflected intensity R, corresponding to the minimum in attenuated total reflection (ATR). The angle of incidence at ATR minimum is called the SPR angle. Now, to analyze the performance of the biosensor, it is necessary to consider the following metrics; particularlly, to optimize the biosensor, we mainly focus on the sensitivity enhancement concerning conventional biosensors (i.e., P/Ag/M) (here, N is the N-parameter to be optimized) expressed as

(2) $${\Delta S}_{RI}^{N}={(S}_{RI}^{N}-S_{RI}^{0})/S_{RI}^{0}$$

The sensitivity to the refractive index change can be expressed as

(3) $$S_{RI}^{N}=\Delta \theta /\Delta n$$

The parameter Δθ represents the angle variation. The detection accuracy (DA) (i.e., signal-to-noise ratio) can be expressed in terms Δθ and full width at half maximum (FWHM) as

(4) DA=Δθ/FWHM

Finally, quality factor (QF) can be expressed in terms of S and FWHM as

(5) $$QF=S_{RI}^{N}/FWHM$$

## 3. Results and Discussions

In this section, we present the optimization results for the SPR biosensor based on the configurations outlined in Table S1. These configurations incorporate various combinations of materials such as Si_3_N_4_, MoS_2_, and ssDNA to evaluate their impact on sensor performance. The optimization process specifically examines how the thickness of the silver layer and other involved layers influence critical parameters, including attenuation, FWHM, and sensitivity enhancement. Notably, Sys_0_, representing the most basic configuration in water (P/Ag/M_H_2_O_), serves as the baseline for comparison and the starting point for optimizing advanced configurations. By systematically modifying the materials and their arrangement, the study aims to identify the best-performing SPR biosensor configuration for virus detection.

### 3.1. Study of the Different Configurations

**Figure 2:**
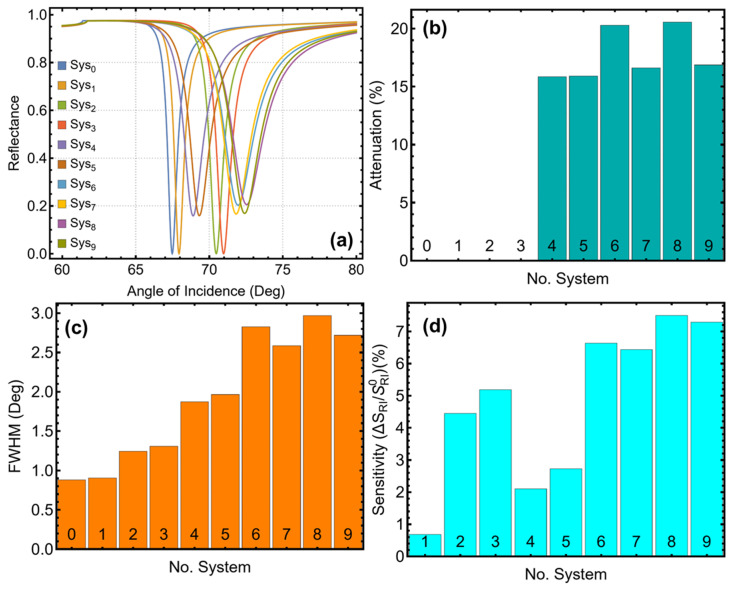
Analysis of SPR biosensor performance for different configurations studied in this work. (**a**) Reflectance curves as a function of the angle of incidence for all systems, highlighting the variation in SPR dip characteristics based on the materials used. (**b**) Attenuation percentage, showing the efficiency of light confinement for each configuration. (**c**) Full-width at half maximum (FWHM) of the SPR curves, indicating the sharpness of the resonance peaks and overall resolution. (**d**) Sensitivity enhancement relative to the baseline configuration (Sys_0_), emphasizing the impact of advanced materials on improving detection capabilities. These results demonstrate the progression from the basic system in water (Sys_0_) to configurations with enhanced sensitivity and optimized SPR properties.

The reflectance curves in Figure 2a illustrate the evolution of SPR characteristics across all configurations, starting from the baseline system, Sys_0_, composed of a prism/silver/medium (P/Ag/M_H_2_O_) structure. Sys_0_ exhibits a broad and shallow resonance dip, reflecting limited plasmonic coupling and weak sensitivity to refractive index changes. As advanced materials such as single-layer MoS_2_, Si_3_N_4_, and ssDNA are introduced in subsequent configurations, the resonance dips become sharper and deeper. This progression is particularly evident in Sys_8_ and Sys_9_, where the integration of Si_3_N_4_ and single-layer MoS_2_ significantly improves plasmonic resonance, while the functionalization with ssDNA enhances the biomolecular binding specificity. The sharper resonance dips observed are crucial for precise virus detection, enabling the sensor to differentiate subtle refractive index changes with high resolution.

The attenuation percentages, shown in Figure 2b and Table S3, quantify the energy coupling efficiency between the incident light and surface plasmons. Sys_0_, with an attenuation of 0.023%, reflects minimal plasmonic coupling, whereas Sys_8_ achieves a significantly higher attenuation of 20.55%, comparable to other advanced configurations such as Sys_6_ (20.30%) and Sys_9_ (16.88%). This increase in attenuation signifies stronger plasmonic excitation, a necessary condition for enhancing the sensitivity of the sensor. Despite the higher attenuation, Sys_8_ maintains a practical signal-to-noise ratio, balancing energy coupling with manageable signal loss, making it well-suited for point-of-care diagnostic applications.

The FWHM values, presented in Figure 2c and Table S3, provide insight into the resolution of the SPR biosensor. Narrower FWHM values are desirable for sharper resonance peaks, which improve detection accuracy. While Sys_0_ exhibits the narrowest FWHM (0.88°), its poor sensitivity enhancement renders it impractical for biosensing applications. Sys_8_, with an FWHM of 2.97°, achieves a favorable balance between sharpness and plasmonic confinement. Although its FWHM is slightly broader than that of Sys_7_ (2.59°), Sys_8_ retains sufficient resolution to detect low concentrations of SARS-CoV-2 biomarkers effectively. This slight trade-off in FWHM is justified by the significant gains in sensitivity and plasmonic interactions.

The sensitivity enhancement results, depicted in Figure 2d and Table S3, decisively establish Sys_8_ as the most effective configuration. Sys_8_ achieves the highest sensitivity enhancement of 7.50%, surpassing all other configurations, including Sys_9_ (7.29%) and Sys_6_ (6.64%). This high sensitivity is attributed to the synergistic effects of the materials employed. Single-layer MoS_2_ provides a high surface area and strong optical properties, enhancing light–matter interactions at the sensor surface. Si_3_N_4_, as a dielectric layer, facilitates efficient propagation of surface plasmons and enables selective functionalization with ssDNA, further improving binding specificity to SARS-CoV-2 biomarkers.

To further emphasize, the systematic progression of configurations, as summarized in Table S1, highlights the deliberate optimization of materials to enhance sensor performance. Starting from Sys_0_, which serves as the baseline, each subsequent configuration introduces additional layers or materials to address specific limitations. For instance

- The addition of Si_3_N_4_ in Sys_2_ improves plasmonic propagation, resulting in increased sensitivity (4.45%) compared to Sys_0_;
- The inclusion of single-layer MoS_2_ in Sys_4_ amplifies light–matter interactions, resulting in enhanced sensitivity (6.64%);
- Sys_8_, which combines Si_3_N_4_, single-layer MoS_2_, and ssDNA, achieves the best overall performance, demonstrating the potential of these materials to elevate biosensor capabilities.

Then, Sys_8_ stands out as the optimal configuration due to its general improvement across all key performance metrics. Unlike other configurations that shine in isolated parameters, Sys_8_ provides a well-balanced combination of high sensitivity (7.50%), practical attenuation (20.55%), and sufficient resolution (2.97°). These attributes make Sys_8_ particularly suitable for real-world applications, where reliability, precision, and ease of use are critical for point-of-care diagnostics.

### 3.2. Silver Thickness Analysis

**Figure 3:**
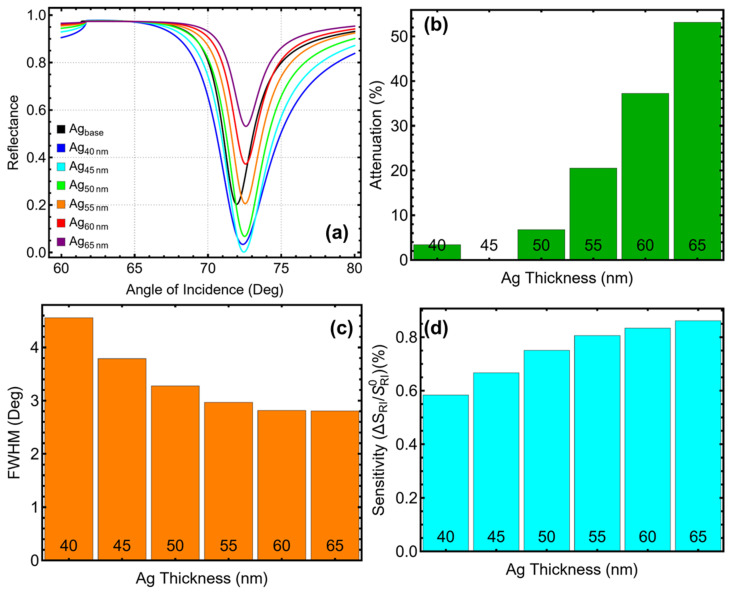
Optimization of silver layer thickness based on Sys_8_. (**a**) Reflectance curves as a function of the angle of incidence for different Ag thicknesses (40–65 nm) in PBS solution, compared to the baseline system (Ag_base_) with initial parameters from Table S2 in water. (**b**) Attenuation percentage, indicating the efficiency of light confinement for each Ag thickness. (**c**) Full-width at half maximum (FWHM) of the SPR resonance curves, highlighting the trade-off between resolution and plasmonic confinement as the Ag thickness increases. (**d**) Sensitivity enhancement relative to the baseline system, showing improved sensitivity with increasing Ag thickness.

The optimization of the silver (Ag) layer thickness is a critical parameter in the performance of the SPR biosensor, as it directly affects key metrics such as sensitivity enhancement, resolution (FWHM), and energy coupling efficiency (attenuation). The results of this optimization are presented in Figure 3 and Table S4, which evaluate the Sys_8_ configuration immersed in PBS solution with silver thicknesses ranging from 40 nm to 65 nm. Additionally, the baseline system (Ag_base_), defined by the initial parameters in Table S2 and immersed in water, is used as a reference for sensitivity enhancement calculations.

The reflectance curves in Figure 3a reveal a progressive sharpening and deepening of the SPR resonance dip as the silver thickness increases. At 40 nm, the resonance dip is broad and shallow, indicating limited coupling and interaction efficiency. By 65 nm, the dip becomes significantly sharper, reflecting enhanced interaction and plasmonic excitation. However, while higher thicknesses improve the optical response, their impact on other parameters, such as attenuation and sensitivity enhancement, must also be considered. The intermediate performance at 45 nm balances these competing factors, providing a favorable combination of sharp resonance and minimal energy losses.

The results in Figure 3b show that attenuation increases sharply with silver thickness. At 40 nm, attenuation is minimal (3.40%), but it rises significantly as the thickness grows, reaching 53.13% at 65 nm. While higher attenuation improves plasmonic interaction, it introduces challenges, such as increased energy loss, which can compromise signal reliability in practical applications. At 45 nm, the attenuation is notably low (0.17%), ensuring robust signal strength and energy efficiency. This result makes 45 nm a practical choice for maintaining reliable sensor performance.

The FWHM results in Figure 3c highlight the trade-off between resonance sharpness and thickness. Thinner layers like 40 nm result in broader resonance peaks (4.56°), while increasing the thickness to 65 nm narrows the FWHM to 2.81°. However, the narrowing trend becomes less pronounced beyond 45 nm, where the FWHM is already reduced to 3.79°. This resolution is sufficient for accurate detection, avoiding the drawbacks of excessive attenuation observed at higher thicknesses.

The sensitivity enhancement data in Figure 3d confirm the improvement in interaction efficiency as silver thickness increases. Sensitivity reaches a maximum of 0.86% at 65 nm, but the incremental gains diminish after 45 nm, where the enhancement is already 0.67%. This diminishing return suggests that thicker layers offer limited additional benefits while introducing practical challenges, further supporting 45 nm as the optimal thickness for balancing sensitivity and energy efficiency. In addition, the linear relationship between silver thickness and sensitivity enhancement, observed in Figure S1, reinforces these findings. The trend indicates a consistent improvement in the sensor’s refractive index sensitivity with increasing thickness. However, this linear growth is constrained by the steep rise in attenuation at higher thicknesses, as shown in Figure 3b. The trend provides a quantitative basis for confirming that 45 nm offers the best compromise between improving sensitivity and avoiding the limitations associated with higher thicknesses.

Based on these results, 45 nm emerges as the optimal silver thickness for the Sys_8_ configuration in PBS solution. This thickness achieves significant sensitivity enhancement (0.67%) while maintaining a well-defined resonance peak (FWHM = 3.79°) and minimal attenuation (0.17%). These combined attributes ensure the practical usability and robustness of the sensor for applications such as the current study.

### 3.3. Silicon Nitride Thickness Analysis

**Figure 4:**
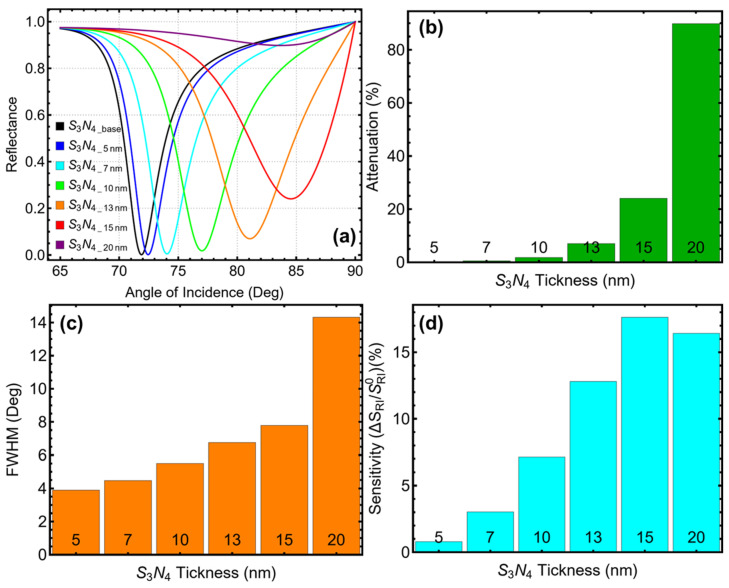
Optimization of silicon nitride (Si_3_N_4_) thickness for the SPR biosensor configuration based on Sys_8_. (**a**) Reflectance curves as a function of the angle of incidence for different Si_3_N_4_ thicknesses (5–20 nm) in PBS solution, compared to the baseline system (Si_3_N_4___base_) with initial parameters from Table S2 in water. (**b**) Attenuation percentage for each thickness. (**c**) Full-width at half maximum (FWHM) of the SPR resonance curves, indicating changes in resolution as the thickness increases. (**d**) Sensitivity enhancement, which is relative to the baseline system.

The results in Figure 4a show that increasing the Si_3_N_4_ thickness from 5 nm to 20 nm causes a shift in the SPR resonance dip toward higher incidence angles. At 5 nm, the resonance dip occurs at approximately 71°, while at 20 nm, the dip shifts to 86°, indicating changes in the interaction between the plasmonic field and the dielectric layer. Thinner Si_3_N_4_ layers allow more effective coupling at lower angles. Still, as the layer thickness increases, the interaction shifts to higher angles due to the altered optical path and refractive index contribution. This progression highlights the dependence of resonance position on the thickness of the dielectric layer, making the choice of thickness critical for optimizing sensor performance.

The attenuation results, shown in Figure 4b and Table S5, demonstrate that increasing the Si_3_N_4_ thickness leads to a rise in energy loss. At 5 nm, the attenuation is minimal (0.17%), reflecting efficient energy utilization with low losses. However, as the thickness increases to 20 nm, the attenuation rises abruptly to 89.80%, indicating substantial energy confinement and high losses that could impair practical sensing applications. At 13 nm, the attenuation is 6.99%, representing an acceptable balance between efficient plasmonic excitation and manageable energy losses. Beyond this thickness, the steep rise in attenuation compromises the sensor’s energy efficiency, making higher thicknesses less desirable for practical use.

The FWHM results in Figure 4c reveal (Table S5) a consistent broadening of the resonance dip with increasing Si_3_N_4_ thickness. At 5 nm, the FWHM is 3.89°, corresponding to a sharp and well-defined resonance peak. This sharpness diminishes as the thickness increases, with the FWHM broadening to 6.76° at 13 nm and further expanding to 14.32° at 20 nm. While broader FWHM values can improve sensitivity to small refractive index changes, they reduce the resolution of the sensor. The intermediate value at 13 nm achieves a favorable balance, offering sufficient resolution for accurate detection while avoiding the excessive broadening seen at higher thicknesses.

The sensitivity enhancement data in Figure 4d (Table S5) and the linear trend in Figure S2 emphasize the systematic improvement in sensitivity with increasing Si_3_N_4_ thickness. At 5 nm, sensitivity enhancement is modest (0.80%) but improves significantly to 12.80% at 13 nm. Beyond this thickness, sensitivity peaks at 17.64% for 15 nm but declines slightly to 16.42% at 20 nm. This diminishing return suggests that further increases in thickness beyond 13 nm offer marginal benefits while introducing trade-offs in attenuation and FWHM. The linear fit in Figure S2 confirms the proportional relationship between thickness and sensitivity within the range studied, reinforcing the choice of 13 nm as a practical compromise.

To emphasize, at 13 nm, the Si_3_N_4_ layer achieves optimal performance across all metrics. The sensitivity enhancement of 12.80% represents a significant improvement, while the FWHM of 6.76° ensures reliable resolution. The attenuation remains at an acceptable level of 6.99%, enabling efficient energy coupling without excessive losses. These combined attributes make 13 nm the most suitable thickness for Si_3_N_4_ in the current SPR biosensor configuration, balancing sensitivity, resolution, and energy efficiency for practical diagnostic applications.

### 3.4. MoS_2_ Layers Analysis

**Figure 5:**
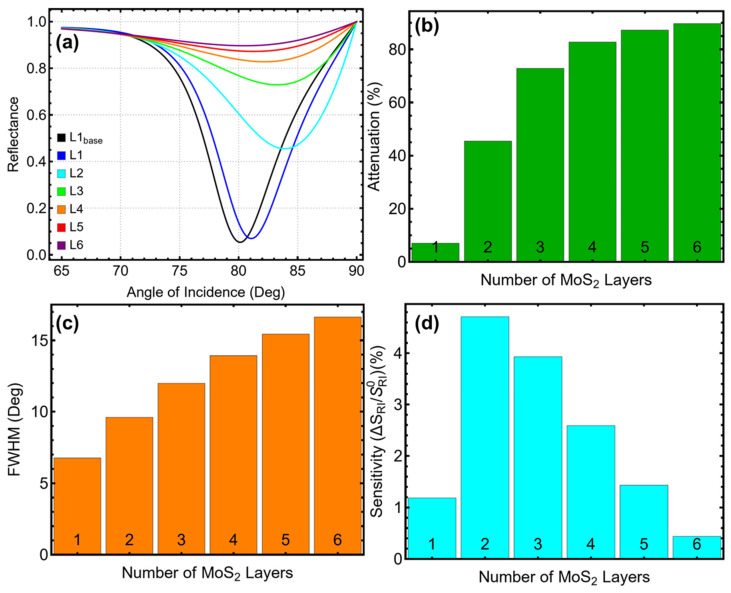
Optimization of the number of MoS_2_ layers for the SPR biosensor. (**a**) Reflectance curves as a function of the angle of incidence for systems with varying numbers of MoS_2_ layers (1–6) in PBS solution, compared to the baseline system (L_1base_) with optimized silver and silicon nitride thicknesses in water. (**b**) Attenuation percentage, showing the impact of increasing MoS_2_ layers on energy confinement. (**c**) Full-width at half maximum (FWHM) of the SPR resonance curves, indicating changes in resolution with additional MoS_2_ layers. (**d**) Sensitivity enhancement relative to the baseline system, showing the effect of the number of MoS_2_ layers on sensor sensitivity.

The reflectance curves in Figure 5a exhibit a systematic shift in the SPR resonance dip toward higher incidence angles as the number of MoS_2_ layers increases from 1 to 6. For the baseline system with a single MoS_2_ layer (L_1base_), the resonance dip occurs at approximately 80°, with a well-defined and sharp profile. Adding additional layers progressively shifts the resonance to higher angles, reaching approximately 85° for 6 layers (L_6_). While this shift reflects enhanced interaction between the plasmonic field and the MoS_2_ layers, the curves also become broader and less pronounced with more layers, which reduces the resolution of the sensor.

The attenuation data presented in Figure 5b (Table S6) reveal a significant increase with additional MoS_2_ layers. For a single layer, attenuation is minimal at 6.99%, ensuring strong signal retention. When two layers are added, attenuation increases to 45.50%, a level that remains acceptable for practical applications. However, the addition of more layers leads to a steep rise in attenuation, with values of 72.89% for three layers and 89.66% for six layers. This rapid increase highlights the substantial energy loss associated with excessive layers, which diminishes the practicality of configurations beyond two layers.

The FWHM results shown in Figure 5c (Table S6) emphasize the trade-offs associated with increasing the number of MoS_2_ layers. At 1 layer, the FWHM is 6.76°, indicating a sharp resonance peak and good resolution. Increasing to two layers broadens the FWHM moderately to 9.61°, maintaining adequate resolution for sensing applications. However, the FWHM continues to broaden significantly with additional layers, reaching 11.98° for three layers and 16.64° for six layers. This broadening indicates reduced precision in detecting refractive index changes, which compromises the sensor’s overall accuracy when more than two layers are used.

The sensitivity enhancement results in Figure 5d (Table S6) show that the sensor achieves maximum enhancement with two MoS_2_ layers, where the improvement reaches 4.71%, compared to 1.19% for a single layer. This enhancement reflects a stronger interaction between the plasmonic field and the analyte. However, as more layers are added, sensitivity declines, dropping to 3.93% for three layers and decreasing further to 0.44% for six layers. The decline is likely due to increased optical losses and diminished efficiency of light–matter interaction as the number of layers increases. The linear trend observed in Figure S3 further supports the diminishing sensitivity beyond two layers.

The configuration with 2 MoS_2_ layers achieves the best balance among the competing metrics. At this thickness

- Sensitivity enhancement is maximized at 4.71%, providing a substantial improvement over the baseline;
- Attenuation remains manageable at 45.50%, avoiding the excessive losses observed with additional layers;
- The FWHM of 9.61° ensures sufficient resolution without the excessive broadening seen with higher layer numbers.

These results demonstrate the importance of careful optimization of the number of MoS_2_ layers in SPR biosensors. While increasing the layers initially enhances sensitivity and interaction with the plasmonic field, excessive layers introduce energy losses and reduce resolution. The selection of two MoS_2_ layers provides a balanced configuration, enabling high sensitivity, manageable attenuation, and reliable resolution. This optimization is essential for ensuring the biosensor’s performance and usability in sensing applications.

### 3.5. Thiol-Tethered ssDNA Thickness Analysis

**Figure 6:**
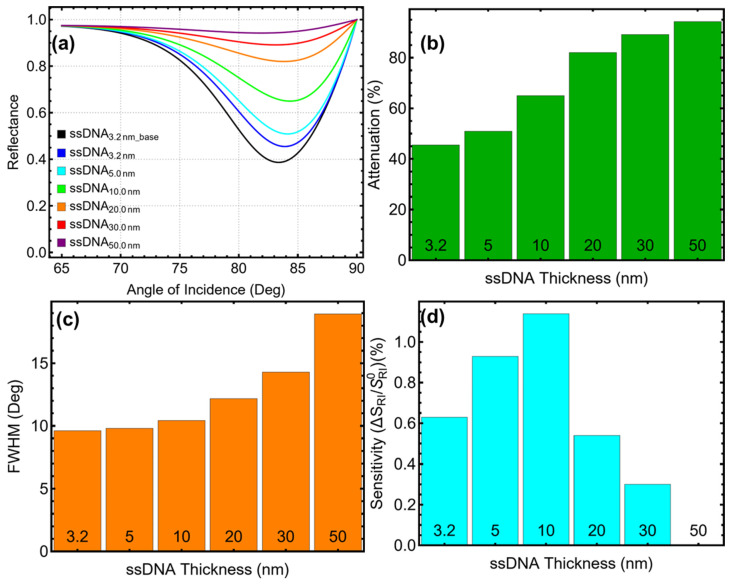
Optimization of ssDNA layer thickness for the SPR biosensor. (**a**) Reflectance curves as a function of the angle of incidence for varying ssDNA thicknesses (3.2–50 nm) in PBS solution, compared to the baseline system (ssDNA_3.2nm_base_) with the initial ssDNA parameters, optimized silver and silicon nitride thicknesses, and two MoS_2_ layers in water. (**b**) Attenuation percentage, demonstrating the effect of increasing ssDNA thickness on energy confinement. (**c**) Full-width at half maximum (FWHM) of the SPR resonance curves, highlighting changes in resolution as the ssDNA layer thickness increases. (**d**) Sensitivity enhancement relative to the baseline system, showing the relationship between ssDNA thickness and biosensor sensitivity.

The optimization of the ssDNA layer thickness significantly impacts the performance of the SPR biosensor, as observed in Figure 6 and Figure S4, and Table S7. The reflectance curves in Figure 6a indicate a progressive shift of the resonance dip toward higher incidence angles with increasing ssDNA thickness. The baseline system (ssDNA_3.2nm_base_) with a thickness of 3.2 nm exhibits a resonance dip at approximately 83°, and as the thickness increases to 50 nm, the dip shifts to 85°. While the shift suggests enhanced optical path length due to the thicker layers, the resonance dip broadens significantly at higher thicknesses, reducing sensor resolution.

The attenuation results in Figure 6b reveal a steady increase with thicker ssDNA layers. At 3.2 nm, attenuation is 45.50%, reflecting efficient energy coupling with acceptable losses. Increasing the thickness to 5 nm raises attenuation moderately to 50.88%, which remains practical for biosensor applications. Beyond this value, attenuation escalates sharply to 64.97% for 10 nm and peaks at 94.22% for 50 nm. These higher values indicate substantial energy losses, which compromise the sensor’s performance in realistic sensing applications. The manageable attenuation at 5 nm supports its selection as the optimal thickness.

The FWHM results in Figure 6c show an increasing trend with thicker ssDNA layers, reflecting a reduction in the sharpness of the resonance dip. For the baseline system, the FWHM is 9.61° and it broadens slightly to 9.79° at 5 nm, maintaining sufficient resolution for biosensing applications. As the thickness increases further, the FWHM broadens considerably, reaching 12.17° at 20 nm and 18.93° at 50 nm. The broader resonance peaks observed at higher thicknesses reduce the precision of the sensor, making configurations with thicker ssDNA layers less desirable.

The sensitivity enhancement results in Figure 6d and Figure S4 demonstrate that the sensor achieves maximum enhancement at 5 nm, with a value of 0.93%, compared to 0.63% for the baseline configuration. Beyond 5 nm, sensitivity declines, dropping to 1.14% at 10 nm and falling to zero at 50 nm. The linear trend in Figure S4 indicates diminishing sensitivity enhancement with increasing ssDNA thickness. This trend suggests that while thicker ssDNA layers can increase optical interaction, they also introduce excessive optical losses that deny any sensitivity gains.

The choice of 5 nm as the optimal ssDNA thickness is justified by its balanced performance across key metrics:

- Sensitivity enhancement is maximized at 0.93%, providing a meaningful improvement over the baseline configuration;
- Attenuation remains moderate at 50.88%, avoiding the steep losses observed at higher thicknesses;
- The FWHM of 9.79° ensures adequate resolution, maintaining the precision required for biosensing applications.

These results highlight the importance of optimizing the ssDNA thickness to balance sensitivity, resolution, and energy efficiency. Thinner layers, such as 3.2 nm, offer sharper resonance and lower attenuation but lack sufficient sensitivity enhancement. Conversely, thicker layers introduce significant energy losses and reduced resolution, diminishing the sensor’s practical usability. The 5 nm configuration achieves a favorable balance, advancing the development of SPR biosensors for reliable molecular detection.

### 3.6. The Optimized SRP Biosensor for the SARS-CoV-2 Sensing

Table 1 outlines the optimized parameters for the Sys_8_ configuration (Table S1), including the refractive index and thickness values of each material layer, along with the refractive index of SARS-CoV-2 RNA at varying concentrations in PBS. These optimized values reflect a biosensor design tailored to balance sensitivity, resolution, and attenuation for precise viral detection. The optimized silver layer thickness of 45.0 nm ensures efficient plasmonic resonance, while the 13.0 nm silicon nitride (Si_3_N_4_) layer enhances dielectric coupling and supports surface plasmon propagation. The addition of 2 MoS_2_ layers (1.30 nm thickness) improves the interaction between the plasmonic field and the analyte, leveraging the material’s high surface area and biocompatibility. Furthermore, the 5.0 nm ssDNA layer provides specific binding sites for SARS-CoV-2 RNA, achieving selectivity while maintaining manageable energy losses.

SARS-CoV-2 RNA in PBS exhibits refractive index values ranging from 1.340 (150 mM) to 1.355 (525 mM), corresponding to varying viral concentrations. These values have been taken from Kumar et al. [25], which demonstrated a direct relationship between refractive index and viral concentration in SPR systems. The incremental refractive index shifts enable the biosensor to distinguish between different concentrations of viral RNA, validating its capability for sensitive and quantitative detection. Additionally, the refractive index of PBS, measured as 1.334, serves as a baseline for evaluating changes induced by the presence of SARS-CoV-2 RNA.

**Figure 7:**
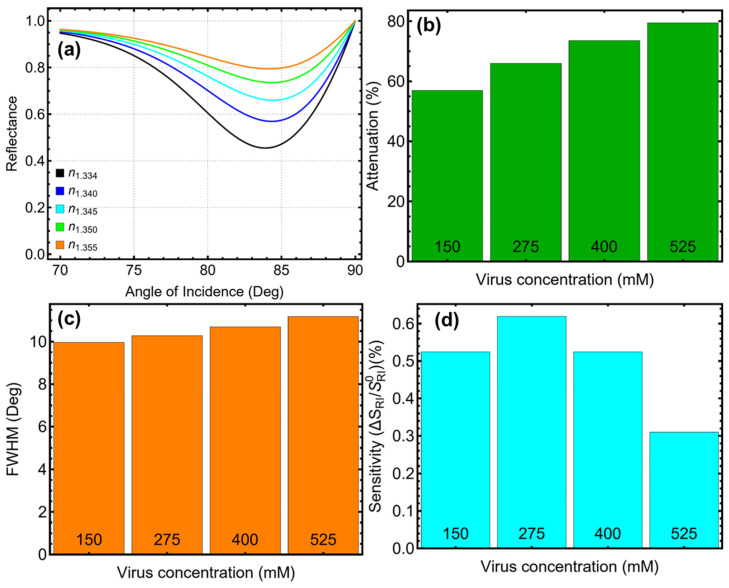
Performance of the optimized SPR biosensor for SARS-CoV-2 detection at varying viral concentrations. (**a**) SPR reflectance curves as a function of the angle of incidence, showing shifts in the resonance dip due to changes in refractive index at different SARS-CoV-2 concentrations. (**b**) Attenuation percentage, highlighting the effect of viral concentration on energy confinement within the biosensor. (**c**) Full-width at half maximum (FWHM) of the SPR resonance curves, demonstrating the resolution changes as the virus concentration increases. (**d**) Sensitivity enhancement relative to the baseline system (n = 1.334 in PBS), validating the biosensor’s capacity to detect and differentiate SARS-CoV-2 concentrations through refractive index variations.

To stress, the performance of the optimized SPR biosensor for SARS-CoV-2 detection was evaluated across viral concentrations ranging from 150 mM to 525 mM, with the results presented in Figure 7 and Figure S5, and Table 2. The sensor’s ability to detect changes in refractive index due to viral adsorption was demonstrated by analyzing the reflectance behavior, attenuation, resolution, and sensitivity enhancement.

Reflectance behavior, as shown in Figure 7a, highlights a consistent shift in the resonance dip toward higher angles with increasing viral concentration. The baseline system in PBS (n = 1.334) exhibits a resonance dip at approximately 83°, which shifts progressively to 86° at the highest concentration of 525 mM (n = 1.355). This angular shift directly correlates with the incremental refractive index changes caused by higher viral concentrations. The ability to track these shifts underscores the sensor’s effectiveness in detecting SARS-CoV-2 at varying concentrations, providing a robust basis for its diagnostic application.

The attenuation results, depicted in Figure 7b and Table 2, reveal a gradual increase in energy losses as the virus concentration rises. Starting at 56.93% for 150 mM, attenuation increases to 79.41% at 525 mM. While greater optical interaction at higher concentrations results in improved refractive index detection, the corresponding increase in attenuation reflects a trade-off between enhanced sensitivity and energy efficiency. Nevertheless, the observed attenuation values remain within acceptable limits for practical biosensor performance, ensuring its applicability even at higher viral concentrations.

The impact of increasing concentration on the sensor’s resolution is evident from the FWHM trends shown in Figure 7c. The FWHM begins at 9.96° for the baseline system and broadens slightly to 11.18° at the highest viral concentration. This broadening reflects the cumulative effects of refractive index changes and optical losses, which marginally reduce the sharpness of the resonance peak. However, the FWHM values remain sufficiently narrow to maintain reliable resolution for SARS-CoV-2 detection, ensuring the sensor’s practicality in diagnostic applications.

On the other hand, the biosensor’s sensitivity enhancement, as shown in Figure 7d, peaks at 0.62% for a viral concentration of 275 mM before declining at higher concentrations. At the lowest concentration (150 mM), the enhancement is 0.52%, decreasing to 0.31% at 525 mM. The linear trend in Figure S5 suggests diminishing sensitivity improvements at higher concentrations, likely due to saturation effects and increased attenuation. These findings indicate that the biosensor performs optimally in the low-to-moderate concentration range, a critical requirement for early-stage detection of SARS-CoV-2 in clinical settings.

**Figure 8:**
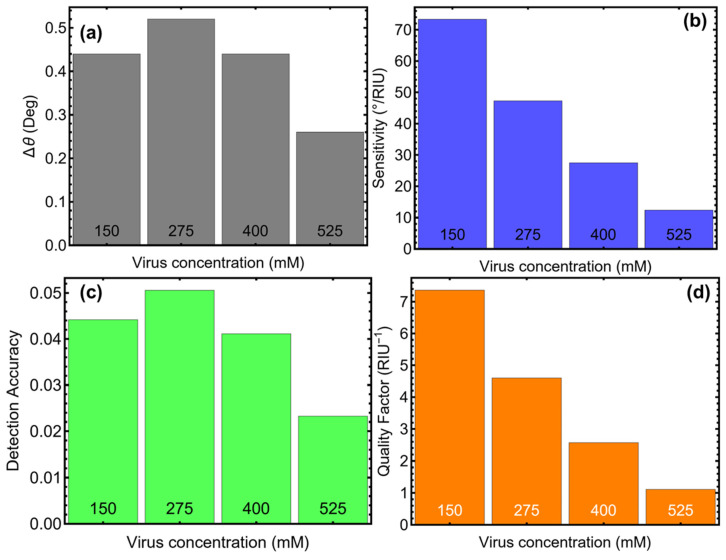
Key performance metrics of the optimized SPR biosensor for SARS-CoV-2 detection at varying viral concentrations. (**a**) Angular variation (Δθ), indicating the shift in the resonance angle corresponds to refractive index changes induced by viral adsorption. (**b**) Sensitivity to refractive index changes (°/RUI), highlighting the biosensor’s responsiveness to varying SARS-CoV-2 concentrations. (**c**) Detection accuracy, representing the precision of resonance angle determination under different viral loads. (**d**) Quality factor (RIU^−1^), illustrating the balance between sensitivity and resolution as a function of virus concentration.

The sensing performance of the optimized SPR biosensor was evaluated using key metrics, including angle variation (Δθ), sensitivity to refractive index change (S), detection accuracy (DA), and quality factor (QF), as shown in Figure 8 and Table 3. These metrics provide a comprehensive understanding of the biosensor’s capabilities across different concentrations of SARS-CoV-2.

Then, the angular variation (Δθ), depicted in Figure 8a, reflects the shift in the resonance angle caused by changes in the refractive index due to viral adsorption. At a concentration of 150 mM, Δθ is 0.44°, increasing slightly to 0.52° at 275 mM, indicating a strong angular response to viral adsorption at these concentrations. However, as the viral concentration increases to 400 mM and 525 mM, Δθ decreases to 0.44° and 0.26°, respectively. This reduction at higher concentrations suggests a saturation effect where additional viral adsorption has a diminishing impact on the resonance angle. These results further emphasize the sensor’s high sensitivity at lower concentrations, making it ideal for early detection of SARS-CoV-2.

Sensitivity (S), as shown in Figure 8b, quantifies the angular response per unit change in refractive index. At 150 mM, sensitivity reaches its highest value of 73.33°/RIU, demonstrating the biosensor’s strong responsiveness at low viral loads. As concentration increases, sensitivity decreases to 47.27°/RIU at 275 mM and drops further to 12.38°/RIU at 525 mM. This trend aligns with the observed decline in angular variation and reflects the biosensor’s reduced ability to detect smaller refractive index changes at higher concentrations. These findings confirm that the biosensor is most effective for detecting early or moderate levels of viral presence, where sensitivity is critical.

Detection accuracy (DA), shown in Figure 8c, measures the precision of the biosensor in determining resonance angles. The highest DA value of 0.05 is observed at 275 mM, suggesting the best balance between angular shifts and signal clarity at moderate viral concentrations. At 150 mM, DA is slightly lower at 0.04, reflecting strong but slightly less precise detection at low concentrations. However, DA decreases significantly to 0.02 at 525 mM due to broader resonance curves caused by higher viral loads. These results suggest that the biosensor achieves optimal precision at moderate concentrations, further supporting its utility in practical diagnostic applications.

The quality factor (QF), which represents the trade-off between sensitivity and resolution, is illustrated in Figure 8d. At 150 mM, QF is 7.36, the highest among all tested concentrations, highlighting the biosensor’s ability to detect low viral loads with high resolution. The QF decreases to 4.60 at 275 mM and continues to decline to 2.57 at 400 mM and 1.11 at 525 mM. These results underscore the biosensor’s strong performance at low concentrations, while its resolution diminishes as viral concentration increases. The high QF at low concentrations positions the biosensor as a reliable tool for early detection, where precision and sensitivity are paramount.

### 3.7. Sensing SARS-CoV-2 at Very-Low Concentrations

The results presented in Figure S6 and Table S8 provide a detailed assessment of the performance of the proposed SPR biosensor for SARS-CoV-2 detection at very low virus concentrations ranging from 0.01 to 10 mM (obtained by extrapolating the experimental results observed in [25]). This evaluation highlights the limitations and capabilities of the biosensor, particularly in terms of its sensitivity to refractive index changes induced by viral adsorption.

The SPR reflectance curves in Figure S6a demonstrate minimal shifts in the resonance angle across all tested concentrations. These findings are corroborated by the attenuation values shown in Figure S6b, which exhibit only slight variations, particularly at concentrations below 1 mM. The FWHM values (Figure S6c) remain largely unchanged, indicating a consistent resolution of the resonance curves regardless of the virus concentration. Notably, the sensitivity enhancement depicted in Figure S6d reveals that the biosensor starts exhibiting measurable sensitivity to virus presence at concentrations of 1 mM and above, with the sensitivity enhancement increasing to 0.05% at 10 mM. This trend reflects the refractive index change threshold required to activate the biosensor’s response, emphasizing its limitation in detecting concentrations below 1 mM.

Figure S7 presents a linear fit of the sensitivity enhancement data, reinforcing the observed correlation between the virus concentration and sensitivity. However, the slope of the linear trend highlights the minimal enhancement observed at extremely low concentrations (below 1 mM). This limitation is further evident in Table S8, where the refractive index values of SARS-CoV-2 in PBS remain nearly constant for concentrations below 1 mM. The biosensor’s attenuation and FWHM metrics also exhibit negligible variation in this range, further validating the minimal response at these concentrations. The proposed biosensor demonstrates a promising detection threshold, with its sensitivity becoming meaningful at concentrations of 1 mM and higher. While this performance may not meet the requirements for clinical applications where detection of viral concentrations below 1 mM is critical, it underscores the biosensor’s utility in non-clinical settings, such as environmental monitoring or detecting higher viral loads in research applications. The inability to detect lower concentrations is primarily attributed to the refractive index sensitivity limitations, which could be addressed in future designs by incorporating additional amplification techniques or signal-enhancing layers.

**Figure 9:**
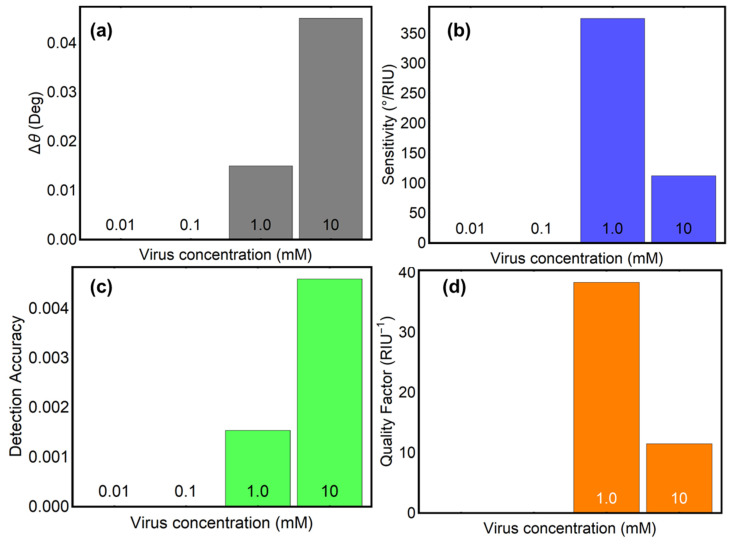
Key performance metrics of the optimized SPR biosensor for SARS-CoV-2 detection at varying very-low viral concentrations from 0.01 to 10 mM. (**a**) Angular variation (Δθ). (**b**) Sensitivity to refractive index changes (°/RUI). (**c**) Detection accuracy. (**d**) Quality factor (RIU^−1^).

On the other hand, the results obtained at very low SARS-CoV-2 concentrations (0.01, 0.1, 1.0, and 10 mM) confirm that the biosensor begins to respond reliably at 1.0 mM, as shown in Figure 9 and Table 4. In particular, the angular variation (Δθ) results, depicted in Figure 9a, show zero resonance shifts for concentrations below 1.0 mM, reflecting the challenges in detecting extremely low viral loads. However, at 1.0 mM, a measurable Δθ of 0.02° is observed, increasing to 0.05° at 10 mM. This indicates that the biosensor is capable of detecting changes in the refractive index at millimolar concentrations, albeit with a higher detection limit compared to clinical requirements. These findings suggest that the sensor design is fundamentally sound but requires refinement to achieve the sensitivity needed for nanomolar or picomolar concentrations typical of clinical diagnostics.

The sensitivity to refractive index changes (S), presented in Figure 9b, peaks at 1.0 mM with a remarkable value of 375.01°/RIU. This performance is higher than that observed at concentrations tested above, such as 150 or 275 mM, demonstrating the sensor’s efficacy in detecting low viral loads. However, the absence of response at concentrations below 1.0 mM highlights the need for additional engineering to enhance the sensor’s interaction with the target analyte and improve its sensitivity in clinically relevant ranges.

Detection accuracy (DA) and quality factor (QF), shown in Figure 9c,d, further validate the biosensor’s performance at millimolar concentrations. At 1.0 mM, DA reaches 0.002, and QF achieves an impressive value of 38.34 RIU^−1^. These metrics indicate a high degree of precision and an optimal trade-off between sensitivity and resolution at this concentration. However, the lack of meaningful DA and QF values below 1.0 mM reinforces the reviewer’s observation that the current sensor configuration may not yet be suitable for direct clinical application.

To address this limitation, it is important to note that the current study provides a theoretical foundation and proof of concept for the biosensor. The insights gained from this work can inform future experimental efforts to enhance the sensor’s sensitivity and reduce the detection limit. For instance, integrating additional signal amplification techniques, optimizing surface functionalization, or employing alternative transduction mechanisms could enable the detection of viral loads in the nanomolar or picomolar range, aligning the sensor’s performance with clinical needs.

### 3.8. Experimental Feasibility of the Sys_8_

To highlight, the proposed SPR biosensor demonstrates not only theoretical optimization but also practical feasibility for experimental realization. Each component of the biosensor, from the material selection to the structural configuration, has been designed with consideration for scalable and cost-effective manufacturing techniques.

- The 45 nm silver layer and 13 nm silicon nitride layer, integral to the optimized configuration, can be fabricated using well-established physical vapor deposition (PVD) [28] or chemical vapor deposition (CVD) [29] techniques. These methods are widely used in industrial and research settings, ensuring precise thickness control and reproducibility. The deposition of silver layers with sub-nanometer precision has been demonstrated in numerous studies [30,31], providing confidence in the feasibility of this biosensor’s metallic layer;
- The incorporation of 2 MoS_2_ layers leverages advancements in two-dimensional material synthesis. Methods such as mechanical exfoliation, liquid-phase exfoliation, or chemical vapor deposition are capable of producing high-quality MoS_2_ with excellent uniformity [32]. Furthermore, the transfer of MoS_2_ films onto the biosensor substrate is achievable using well-documented techniques [33], including wet transfer processes, which maintain the integrity of the layered structure and ensure compatibility with the overall SPR biosensor design;
- The thiol-tethered ssDNA recognition layer, optimized at 5 nm thickness, can be efficiently immobilized onto the biosensor surface using thiol-gold chemistry [34]. This method is a standard procedure in biosensor functionalization, offering robust and stable attachment of biomolecules. Additionally, the functionalization process can be scaled for mass production using automated microfluidic systems, ensuring reproducibility and minimizing variability in ssDNA layer deposition;
- The biosensor’s components can be assembled using modular design principles [35], which facilitate the integration of the individual layers into a cohesive platform. Standard cleanroom facilities and assembly techniques employed in microelectronics fabrication can be adapted for the production of the proposed biosensor. The use of commercially available BK7 prisms and established PBS solution preparation further reduces the complexity of manufacturing;
- The experimental feasibility of similar SPR-based biosensors has been demonstrated in recent studies, which successfully employed the Kretschmann configuration for viral detection [36]. These studies serve as experimental validation for the scalability and functional reliability of the proposed biosensor. Moreover, the linear relationship between refractive index changes and viral concentration, as observed in our study, aligns with existing experimental findings, further supporting the feasibility of manufacturing the proposed design.

On the other hand, Table 5 provides a comparative analysis of SPR biosensors with advanced material configurations for detecting biomolecules such as SARS-CoV-2 RNA [37,38,39,40]. The listed configurations demonstrate the diversity of materials employed to enhance SPR sensor performance, evidencing variations in sensitivity depending on the sensor design and material properties. While some sensors achieve extremely high sensitivities, such as the ITO/3-layer Tellurene/10-layer MoS_2_–COOH sensor reporting a sensitivity of 8.4 × 10^4^°/RIU [40], this is attributed to the unique optical and electronic properties of layered tellurene in conjunction with MoS_2_ functionalized for specific applications. However, it is crucial to note that such high sensitivities often come with trade-offs in terms of manufacturability, cost, and application scope, especially in point-of-care diagnostics.

In comparison, our proposed SPR biosensor demonstrates sensitivities of 73.33°/RIU at 150.0 mM SARS-CoV-2 RNA concentration and 375.01°/RIU at 1.0 mM concentration. Although the sensitivity values at high concentrations are modest compared to some reported designs, the proposed biosensor offers a significant advantage in its practical applicability, material simplicity, and integration feasibility. The choice of bilayer MoS_2_ and silicon nitride (Si_3_N_4_) ensures a balance between sensitivity and cost-effective production.

When compared with designs utilizing multi-material configurations such as TiO_2_-Ag-MoSe_2_/Graphene [37] or Ag/PtSe_2_/MoS_2_ [38], our sensor demonstrates superior performance in terms of sensitivity at lower concentrations while focusing on practical manufacturability. Moreover, this work stresses the potential for further improvements in sensitivity through additional experimental optimization, particularly by refining material functionalization for specific biomolecular targets.

## 4. Conclusions

This study presented the development and optimization of an SPR biosensor designed for the detection of SARS-CoV-2, integrating advanced materials and strategic design parameters. Using a systematic approach, we optimized key elements of the biosensor, including silver layer thickness, silicon nitride layer thickness, the number of MoS_2_ layers, and the thickness of the ssDNA recognition layer. Each optimization step revealed significant insights into the interplay between sensitivity, resolution, and attenuation, enabling the identification of an optimal configuration that balances these competing metrics.

The optimized biosensor exhibited superior performance in detecting SARS-CoV-2 in PBS solutions. Specifically, the final configuration consisted of a 45 nm silver layer, a 13 nm silicon nitride dielectric layer, two MoS_2_ layers, and a 5 nm ssDNA layer. This setup demonstrated high sensitivity, particularly at lower viral concentrations, where sensitivity enhancement was most pronounced. Performance metrics, including angular variation (Δθ), sensitivity to refractive index changes (S), detection accuracy (DA), and quality factor (QF), validated the biosensor’s robustness and reliability. At a concentration of 150 mM, the biosensor achieved a Δθ of 0.44°, a sensitivity of 73.33°/RIU, and a QF of 7.36; meanwhile, at a concentration of 1.0 mM, the biosensor achieved a Δθ of 0.02°, a sensitivity of 375.01°/RIU, and a QF of 38.34, highlighting its exceptional capability for early detection of SARS-CoV-2

The study also revealed a saturation effect at higher concentrations, with diminishing shifts in resonance angle and reduced sensitivity as the viral load increased. These findings highlight the biosensor’s strength in detecting low to moderate concentrations of SARS-CoV-2, making it particularly suitable for early diagnosis, where timely intervention is critical. Additionally, the linear relationship between refractive index changes and viral concentration further supports the biosensor’s potential for quantitative diagnostic applications.

As an additional remark, the integration of MoS_2_ and silicon nitride layers into the SPR framework proved critical for enhancing the biosensor’s performance. MoS_2_ provided a high surface area and strong light–matter interaction, improving sensitivity, while silicon nitride contributed to enhanced resolution and propagation of surface plasmons. The use of ssDNA as the recognition element ensured high selectivity toward SARS-CoV-2, further solidifying the biosensor’s diagnostic precision.

Finally, the proposed SPR biosensor offers a promising platform for rapid, sensitive, and reliable detection of SARS-CoV-2. Its capability to operate effectively at low viral concentrations positions it as a valuable tool for early-stage diagnostics and epidemiological monitoring. Future work could extend this design to multiplexed detection of multiple pathogens or adapt the biosensor for point-of-care applications, further broadening its impact in clinical and field settings.

## Figures and Tables

**Table 1:** Optimized parameters of Sys_8_ in PBS solution and refractive index at different SARS-CoV-2 concentrations.

Material	Refractive Index	Thickness (nm)
BK7 (P)	1.5151	---
Ag	0.056253 + 4.2760	45.0
S_3_N_4_ (SN)	2.0394	13.0
Molybdenum Disulfide (MoS_2_)	5.0805 + 1.1723 i	1.30
ssDNA (Thiol-Tethered, T)	1.462	5.00
PBS (M)	1.334	---
SARS-CoV-2 in PBS	1.340 (@150 mM) 1.345 (@275 mM) 1.350 (@400 mM) 1.355 (@525 mM)	---

**Table 2:** Metric values of optimized Sys_8_ configuration after SARS-CoV-2 adsorption at different concentrations.

Concentration (mM)	PBS + SARS-CoV-2	Enhancement (%)	Attenuation %	FWHM
150	1.340	0.52	56.93	9.96
275	1.345	0.62	65.98	10.28
400	1.350	0.52	73.55	10.69
525	1.355	0.31	79.41	11.18

**Table 3:** Performance metrics of optimized Sys_8_ configuration after the SARS-CoV-2 adsorption at different concentrations.

Concentration (mM)	PBS + SARS-CoV-2	Δθ	*S* (°/*RIU*)	DA	QF (*RIU*^−1^)
150	1.340	0.44	73.33	0.04	7.36
275	1.345	0.52	47.27	0.05	4.60
400	1.350	0.44	27.50	0.04	2.57
525	1.355	0.26	12.38	0.02	1.11

**Table 4:** Performance metrics of optimized Sys_8_ configuration after the SARS-CoV-2 adsorption at different low concentrations.

Concentration (mM)	PBS + SARS-CoV-2	Δθ	*S* (°/*RIU*)	DA	QF (*RIU*^−1^)
0.01	1.3340004000000012	0.0	0.0	0.0	0.0
0.10	1.334004000000001	0.0	0.0	0.0	0.0
1.00	1.3340400000000012	0.02	375.01	0.002	38.34
10.0	1.3344000000000011	0.05	112.5	0.005	11.49

**Table 5:** Comparative analysis of SPR biosensors with advanced material configurations for viral detection.

Sensor Configuration	Sensitivity (°/RIU)	Application	Reference
TiO_2_-Ag-MoSe_2_/Graphene	194.0	Virus detection	[37]
Ag/PtSe2/MoS_2_	162.0	Biomolecular detection	[38]
Graphene/MoS_2_/WS_2_/WSe2	194.0	Food safety	[39]
ITO/3-layer Tellurene/10-layer MoS_2_–COOH	8.4 × 10^4^	Spike glycoprotein detection	[40]
Sys_8_@1.0 mM Sys_8_@150 mM	73.33 375.01	SARS-CoV-2	This work

## Data Availability

The original contributions presented in the study are included in the article/Supplementary Materials; further inquiries can be directed to the corresponding author.

## Supplementary Materials

The following supporting information can be downloaded at https://www.mdpi.com/article/10.3390/bios15010021/s1: Figure S1: Linear fit of the sensitivity enhancement data; Figure S2. Linear fit of the sensitivity enhancement data; Figure S3. Linear fit of the sensitivity enhancement data; Figure S4. Linear fit of the sensitivity enhancement data; Figure S5. Linear fit of the sensitivity enhancement data; Figure S6. Performance of the optimized SPR biosensor for SARS-CoV-2 detection at varying viral low concentrations from 0.01 to 10 mM. (a) SPR reflectance curves as a function of the angle of incidence, showing shifts in the resonance dip due to changes in refractive index at different SARS-CoV-2 concentrations. (b) Attenuation percentage, highlighting the effect of viral concentration on energy confinement within the biosensor. (c) Full-width at half maximum (FWHM) of the SPR resonance curves, demonstrating the resolution changes as the virus concentration increases. (d) Sensitivity enhancement relative to the baseline system (n = 1.334 in PBS or 0 mM), validating the biosensor’s capacity to detect and differentiate SARS-CoV-2 concentrations through refractive index variations; Figure S7. Linear fit of the sensitivity enhancement data at very-low virus concentrations; Table S1. The configuration of the systems analyzed in this study; Table S2. Initial parameters of the proposed SPR Biosensor for sensing SARS-CoV-2; Table S3. Metric values of proposed SPR Biosensors; Table S4. Metric values of Sys8 configuration by changing the silver thickness; Table S5. Metric values of Sys8 by changing the silicon nitride thickness; Table S6. Metric values of Sys8 by increasing the number of Molybdenum Disulfide layers; Table S7. Metric values of Sys8 by changing the ssDNA thickness; Table S8. Metric values of optimized Sys8 configuration after SARS-CoV-2 adsorption at very-low virus concentrations.
