# Supplementary material for: SPR Biosensor Based on Bilayer MoS2 for SARS-CoV-2 Sensing
Source: Biosensors (Basel). 2025 Jan 4;15(1):21. doi: 10.3390/bios15010021 (PMC11763928; doi:10.3390/bios15010021)
Supplement: Supplementary file 1 [file biosensors-15-00021-s001.zip › biosensors-3266694-supplementary.pdf]

biosensors

Supplementary

Multilayer SPR Biosensor Based on Bilayer MoS_2 for SARS-CoV-2 sensing

Supplementary Figures

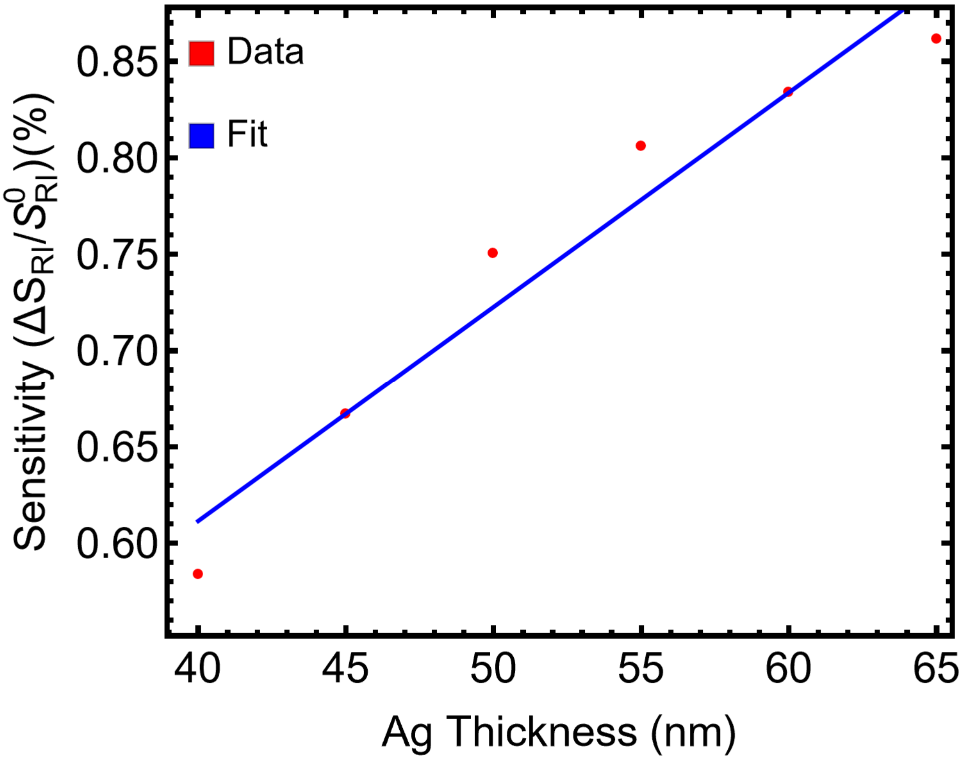

Figure S1. Linear fit of the sensitivity enhancement data.

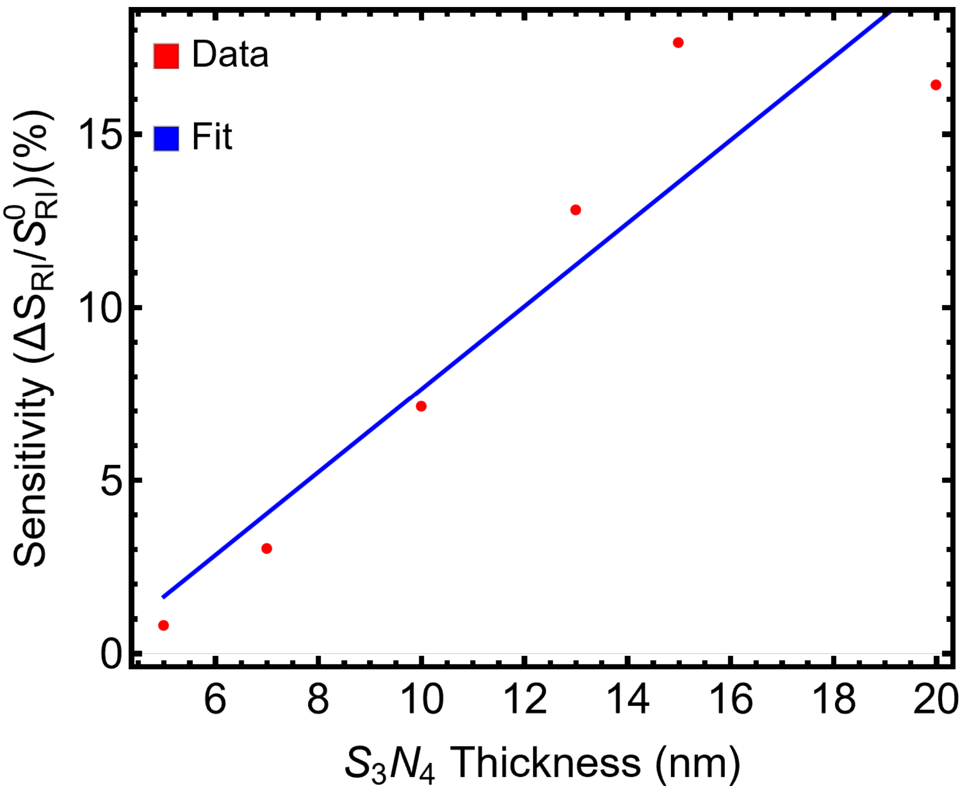

Figure S2. Linear fit of the sensitivity enhancement data.

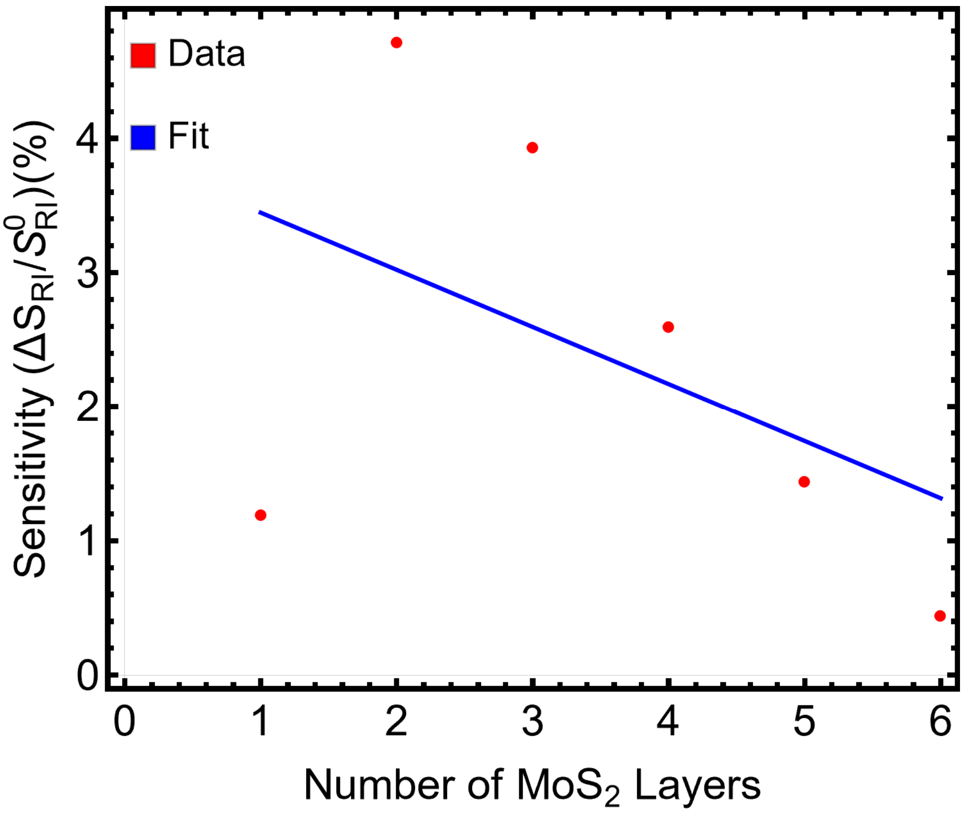

Figure S3. Linear fit of the sensitivity enhancement data.

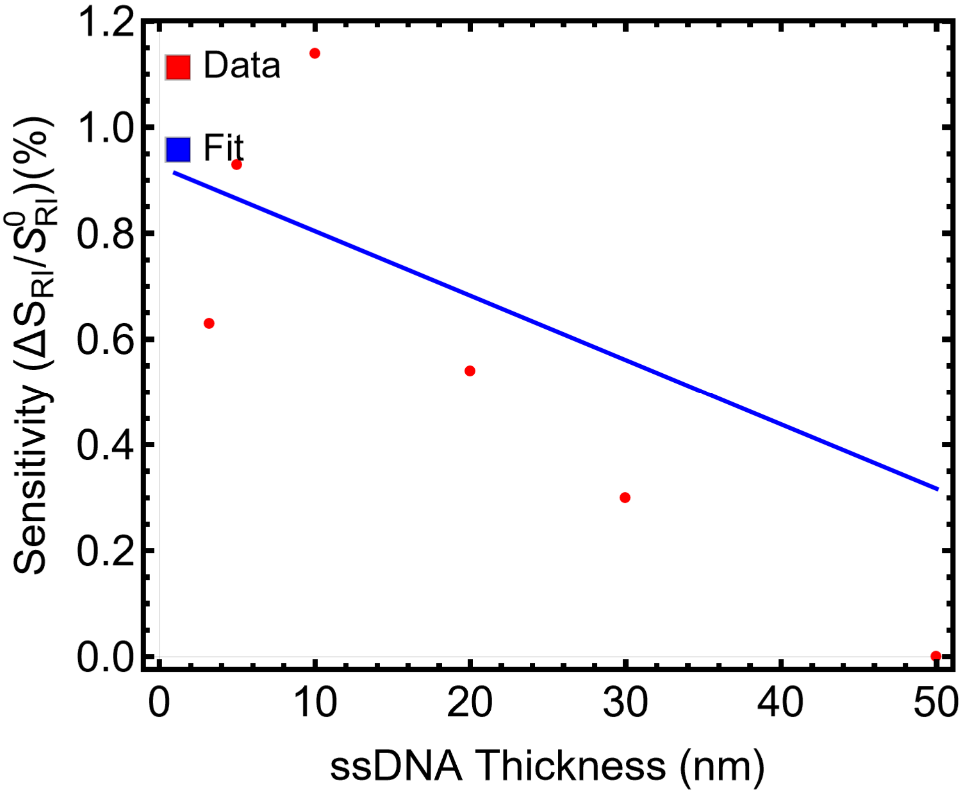

Figure S4. Linear fit of the sensitivity enhancement data.

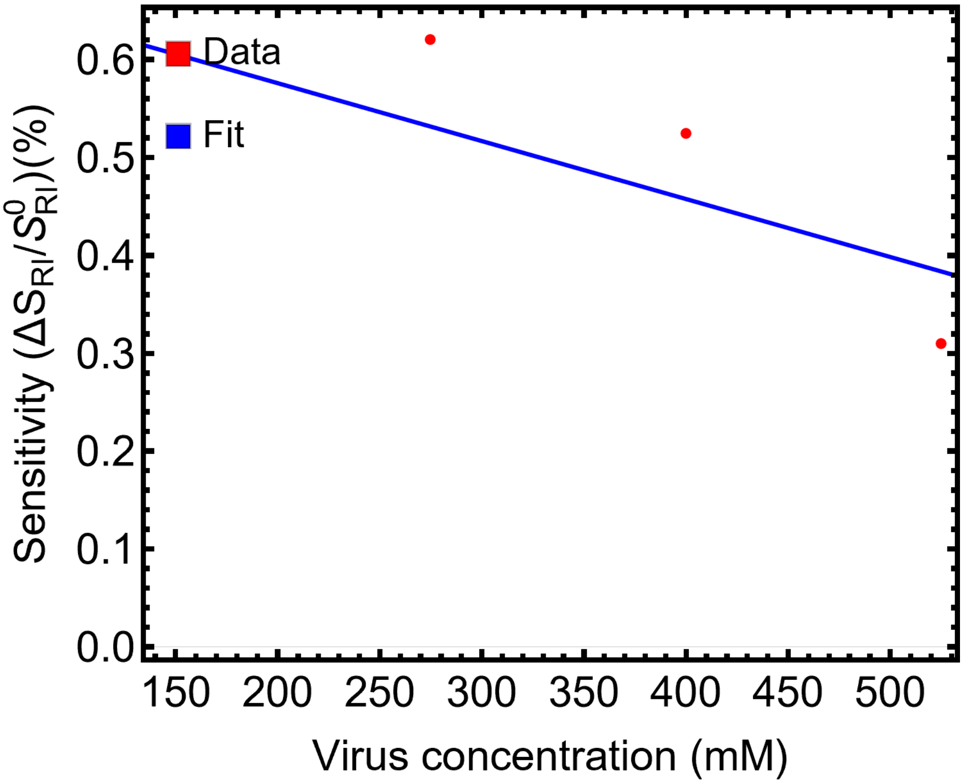

Figure S5. Linear fit of the sensitivity enhancement data.

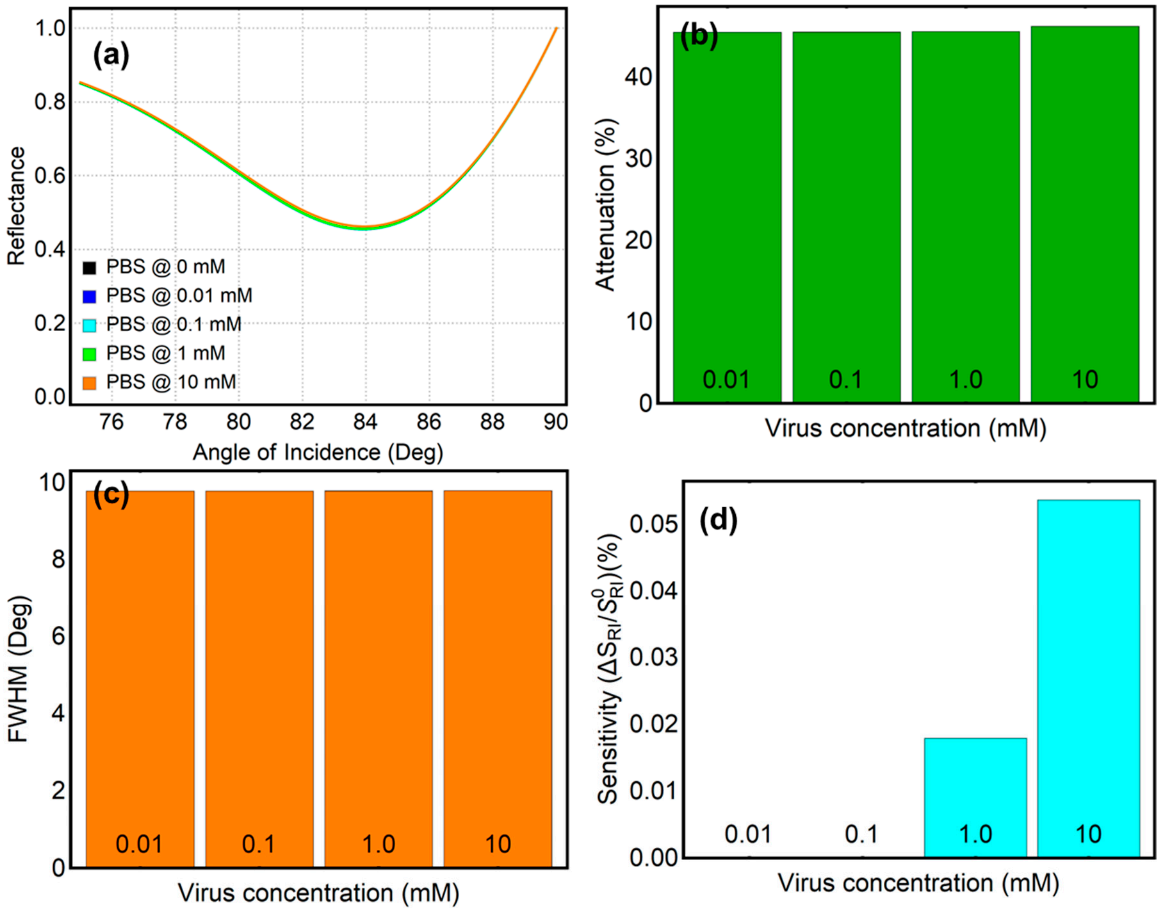

Figure S6. Performance of the optimized SPR biosensor for SARS-CoV-2 detection at varying viral low concentrations from 0.01 to 10 mM. (a) SPR reflectance curves as a function of the angle of incidence, showing shifts in the resonance dip due to changes in refractive index at different SARS-CoV-2 concentrations. (b) Attenuation percentage, highlighting the effect of viral concentration on energy confinement within the biosensor. (c) Full-width at half maximum (FWHM) of the SPR resonance curves, demonstrating the resolution changes as the virus concentration increases. (d) Sensitivity enhancement relative to the baseline system (n = 1.334 in PBS or 0 mM), validating the biosensor's capacity to detect and differentiate SARS-CoV-2 concentrations through refractive index variations.

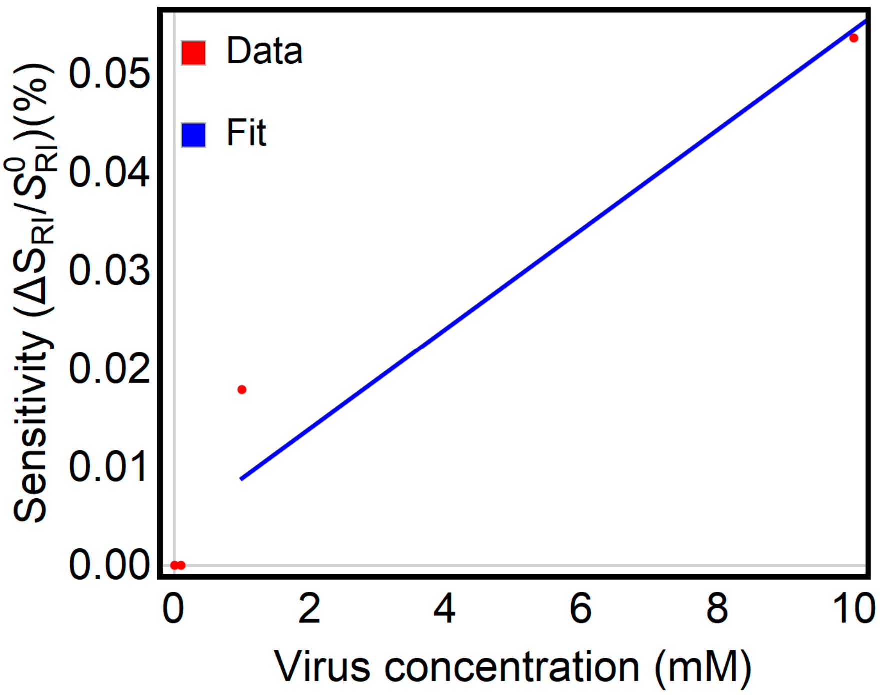

Figure S7. Linear fit of the sensitivity enhancement data at very-low virus concentrations.

Supplementary Tables

Table S1. The configuration of the systems analyzed in this study.

Sys No.	Code	Full Name	Nick Name
1	Sys_0	Prism/Silver/Medium	P/Ag/M_{H2O}
1	Sys_1	Prism/Silver/Medium	P/Ag/M_{PBS}
2	Sys_2	Prism/Silver/S_3N_4/Medium	P/Ag/SN/M_{PBS}
3	Sys_3	Prism/Silver/S_3N_4/ssDNA/Medium	P/Ag/SN/T/M_{PBS}
4	Sys_4	Prism/Silver/Molybdenum Disulfide/Medium	P/Ag/MoS_2/M_{PBS}
5	Sys_5	Prism/Silver/Molybdenum Disulfide/ssDNA/Medium	P/Ag/ MoS_2/T/M_{PBS}
6	Sys_6	Prism/Silver/S_3N_4/Molybdenum Disulfide/Medium	P/Ag/SN/MoS_2/M_{PBS}
7	Sys_7	Prism/Silver/Molybdenum Disulfide/S_3N_4/Medium	P/Ag/MoS_2/SN/M_{PBS}
8	Sys_8	Prism/Silver/S_3N_4/ Molybdenum Disulfide/ssDNA/Medium	P/Ag/SN/MoS_2/T/M_{PBS}
9	Sys_9	Prism/Silver/Molybdenum Disulfide/S_3N_4/ssDNA/Medium	P/Ag/ MoS_2/SN/T/M_{PBS}

Table S2. Initial parameters of the proposed SPR Biosensor for sensing SARS-CoV-2.

Material	Refractive Index	Thickness (nm)	Ref.
BK-7 (P)	1.5151	---	[22]
Silver (Ag)	0.056253 + 4.2760 i	55.0	[23]
S_3N_4 (SN)	2.0394	5.00	[24]
Molybdenum Disulfide (MoS_2)	5.0805 + 1.1723 i	0.34	[25]
ssDNA (Thiol-Tethered, T)	1.462	3.20	[19]
Water (H_2O)	1.33	---	[19]
PBS (M)	1.334	---	[19]

Table S3. Metric values of proposed SPR Biosensors.

Sys No.	Code	Attenuation (%)	FWHM	Sensitivity Enhancement (%)
0	Sys_0	0.023	0.88	0
1	Sys_1	0.023	0.90	0.68
2	Sys_2	0.024	1.24	4.45
3	Sys_3	0.022	1.31	5.19
4	Sys_4	15.84	1.87	2.10
5	Sys_5	15.92	1.97	2.73
6	Sys_6	20.30	2.83	6.64
7	Sys_7	16.62	2.59	6.43
8	Sys_8	20.55	2.97	7.50
9	Sys_9	16.88	2.72	7.29

Table S4. Metric values of Sys_8 configuration by changing the silver thickness.

Thickness (nm)	Attenuation (%)	FWHM	Enhancement (%)
40	3.40	4.56	0.58
45	0.17	3.79	0.67
50	6.75	3.28	0.75
55	20.55	2.97	0.81
60	37.24	2.82	0.83
65	53.13	2.81	0.86

Table S5. Metric values of Sys_8 by changing the silicon nitride thickness.

Thickness (nm)	Attenuation (%)	FWHM	Enhancement (%)
5	0.17	3.89	0.80
7	0.51	4.47	3.03
10	1.82	5.50	7.13
13	6.99	6.76	12.80
15	24.08	7.78	17.64
20	89.80	14.32	16.42

Table S6. Metric values of Sys_8 by increasing the number of Molybdenum Disulfide layers .

Layers	Attenuation (%)	FWHM	Enhancement (%)
L1	6.99	6.76	1.19
L2	45.50	9.61	4.71
L3	72.89	11.98	3.93
L4	82.80	13.93	2.59
L5	87.23	15.46	1.44
L6	89.66	16.64	0.44

Table S7. Metric values of Sys_8 by changing the ssDNA thickness.

Thickness (nm)	Attenuation %	FWHM	Enhancement (%)
3.2	45.50	9.61	0.63
5.0	50.88	9.79	0.93
10.0	64.97	10.42	1.14
20.0	82.01	12.17	0.54
30.0	89.13	14.29	0.30
50.0	94.22	18.93	0.00

Table S8. Metric values of optimized Sys_8 configuration after SARS-CoV-2 adsorption at very-low virus concentrations.

Concentration (mM)	PBS + SARS-CoV-2	Enhancement (%)	Attenuation %	FWHM
0.01	1.3340004000000012	0.0	45.50	9.78
0.10	1.334004000000001	0.0	45.52	9.78
1.00	1.3340400000000012	0.02	45.57	9.78
10.0	1.3344000000000011	0.05	46.23	9.79
